# Supplementary material for: A gustatory receptor tuned to the steroid plant hormone brassinolide in Plutella xylostella (Lepidoptera: Plutellidae)
Source: eLife. 2020 Dec 11;9:e64114. doi: 10.7554/eLife.64114 (PMC7806260; doi:10.7554/eLife.64114)
Supplement: Supplementary file 1. [file elife-64114-supp1.docx]

**Supplementary file 1. Sequence information for gustatory receptors of *Plutella xylostella***

| Reported PxylGrs | PxylGrs in this study | GenBank number | Notes |
| --- | --- | --- | --- |
| PxylGr1^*^ | PxylGr1 | XP_011560061.1 |  |
| PxylGr2^*^ | PxylGr2 | XP_011560059.1 |  |
| PxylGr3^*^ | PxylGr3 | XP_011560063.1 |  |
| PxylGr4^*^ | PxylGr4 | XP_011560065.1 |  |
| PxylGr5^*^ | PxylGr5 | XP_011568607.1 |  |
| PxylGr6^*^ | (delete) |  | Partial sequence of PxylGr5 |
| PxylGr7^*^ | PxylGr7 | XP_011557481.1 |  |
| PxylGr8^*^ | PxylGr8 | XP_011558222.1 |  |
| PxylGr9^*^ | (delete) |  | Partial sequence of PxylGr8 |
| PxylGr10^*^ | PxylGr10 |  |  |
| PxylGr11^*^ | PxylGr11 |  |  |
| PxylGr12^*^ | PxylGr12 |  |  |
| PxylGr13^*^ | PxylGr13 |  |  |
| PxylGr14^*^ | PxylGr14 |  |  |
| PxylGr15^*^ | PxylGr15 |  |  |
| PxylGr16^*^ | PxylGr16 |  |  |
| PxylGr17^*^ | PxylGr17 |  |  |
| PxylGr18^*^ | PxylGr18 |  |  |
| PxylGr19^*^ | PxylGr19 |  |  |
| PxylGr20^*^ | PxylGr20 |  |  |
| PxylGr21^*^ | PxylGr21 |  |  |
| PxylGr22^*^ | PxylGr22 |  |  |
| PxylGr23^*^ | PxylGr23 |  |  |
| PxylGr24^*^ | (delete) |  | Partial sequence of PxylGr19 |
| PxylGr25^*^ | (delete) |  | Repetitive sequence with PxylGr20 |
| PxylGr26^*^ | (delete) |  | Partial sequence of PxylGr21 |
| PxylGr27^*^ | (delete) |  | Repetitive sequence with PxylGr22 |
| PxylGr28^*^ | (delete) |  | Repetitive sequence with PxylGr23 |
| PxylGr29^*^ | PxylGr29 |  |  |
| PxylGr30^*^ | PxylGr30 | XP_011561237.1 |  |
| PxylGr31^*^ | PxylGr31 | XP_011561224.1 |  |
| PxylGr32^*^ | PxylGr32 |  |  |
| PxylGr33^*^ | PxylGr33 |  |  |
| PxylGr34^*^ | PxylGr34 |  |  |
| PxylGr35^*^ | PxylGr35 | XP_011565113.1 |  |
| PxylGr36^*^ | PxylGr36 |  |  |
| PxylGr37^*^ | PxylGr37 | XP_011548483.1 |  |
| PxylGr38^*^ | PxylGr38 | XP_011552106.1 |  |
| PxylGr39^*^ | PxylGr39 |  |  |
| PxylGr40^*^ | PxylGr40 |  |  |
| PxylGr41^*^ | PxylGr41 |  |  |
| PxylGr42^*^ | PxylGr42 |  |  |
| PxylGr43^*^ | PxylGr43 |  |  |
| PxylGr44^*^ | PxylGr44 |  |  |
| PxylGr45^*^ | PxylGr45 |  |  |
| PxylGr46^*^ | PxylGr46 |  |  |
| PxylGr47^*^ | PxylGr47 |  |  |
| PxylGr48^*^ | PxylGr48 |  |  |
| PxylGr49^*^ | PxylGr49 |  |  |
| PxylGr50^*^ | PxylGr50 |  |  |
| PxylGr51^*^ | PxylGr51 |  |  |
| PxylGr52^*^ | PxylGr52 |  |  |
| PxylGr53^*^ | PxylGr53 |  |  |
| PxylGr54^*^ | PxylGr54 |  |  |
| PxylGr55^*^ | PxylGr55 |  |  |
| PxylGr56^*^ | PxylGr56 |  |  |
| PxylGr57^*^ | PxylGr57 |  |  |
| PxylGr58^*^ | PxylGr58 |  |  |
| PxylGr59^*^ | PxylGr59 |  |  |
| PxylGr60^*^ | PxylGr60 |  |  |
| PxylGr61^*^ | PxylGr61 |  |  |
| PxylGr62^*^ | PxylGr62 |  |  |
| PxylGr63^*^ | PxylGr63 |  |  |
| PxylGr64^*^ | PxylGr64 |  |  |
| PxylGr65^*^ | PxylGr65 |  |  |
| PxylGr66^*^ | PxylGr66 |  |  |
| PxylGr67^*^ | PxylGr67 |  |  |
| PxylGr68^*^ | PxylGr68 |  |  |
| PxylGr69^*^ | PxylGr69 |  |  |
| PxylGR1^#^ | (delete) |  | Partial sequence of PxylGr75 |
| PxylGR2^#^ | (delete) |  | Partial sequence of PxylGr34 |
| PxylGR3^#^ | PxylGr72 |  |  |
| PxylGR4^#^ | PxylGr73 |  |  |
| PxylGR5^#^ | (delete) |  | Partial sequence of Gr77 |
| PxylGR6^#^ | (delete) |  | Longer than PxylGr5 |
| PxylGR7^#^ | (delete) |  | Partial sequence of Gr77 |
| PxylGr64f-like^※^ | PxylGr77 | XP_011560052.1 |  |
| PxylGr64f-like^※^ | PxylGr78 | XP_011560051.1 |  |
| PxylGr28b^※^ | PxylGr79 | XP_011565110.1 |  |

*: Named by Engsontia et al., 2014; ^#^: Named by Yang et al., 2017; ^※^: Acquired from the GenBank
