# Supplementary material for: A gustatory receptor tuned to the steroid plant hormone brassinolide in Plutella xylostella (Lepidoptera: Plutellidae)
Source: eLife. 2020 Dec 11;9:e64114. doi: 10.7554/eLife.64114 (PMC7806260; doi:10.7554/eLife.64114)
Supplement: Supplementary file 2. [file elife-64114-supp2.docx]

**Supplementary file 2. Primers used for qPCR, *Xenopus* oocyte expression (Xe), and siRNA synthesis**

| **Purposes** | **Primer Names** | **Sequences (5'–3')** |
| --- | --- | --- |
| qPCR | PxylGr34-qF: | TTTCGTCGAAGCCACGTCACAG |
|  | PxylGr34-qR: | ACCAGCCAATTGTGAACCACCG |
|  | PxylActin-qF: | AACACCGTCATGTCCGGAGGTA |
|  | PxylActin-qR: | ATCTTGATGGTGGAGGGAGCCA |
| Xe | PxylGr34-xeF： | GGATCCgccaccATGCTCGCGCGATTTAAA |
|  | PxylGr34-xeR: | CTCGAGTTACGTAGCAAATTGTAATAG |
| siRNA | GFP-siRNA-Oligo1： | GGATCCTAATACGACTCACTATAGATGGAAACATTCTTGGAC |
|  | GFP-siRNA-Oligo2： | AAGTCCAAGAATGTTTCCATCTATAGTGAGTCGTATTAGGATCC |
|  | GFP-siRNA-Oligo3： | GGATCCTAATACGACTCACTATAGTCCAAGAATGTTTCCATC |
|  | GFP-siRNA-Oligo4： | AAGATGGAAACATTCTTGGACTATAGTGAGTCGTATTAGGATCC |
|  | PxylGr34-siRNA-Oligo1： | GGATCCTAATACGACTCACTATAGACGTCAGTTATTGTACAC |
|  | PxylGr34-siRNA-Oligo2： | AAGTGTACAATAACTGACGTCTATAGTGAGTCGTATTAGGATCC |
|  | PxylGr34-siRNA-Oligo3： | GGATCCTAATACGACTCACTATAGTGTACAATAACTGACGTC |
|  | PxylGr34-siRNA-Oligo4： | AAGACGTCAGTTATTGTACACTATAGTGAGTCGTATTAGGATCC |

F: forward primer; R: reverse primer. The underlined indicate restriction recognition sites, the minuscule indicate Kozak sequence.
